# Whole Genome Scan Reveals Molecular Signatures of Divergence and Selection Related to Important Traits in Durum Wheat Germplasm

**DOI:** 10.3389/fgene.2020.00217

**Published:** 2020-04-21

**Authors:** Francesca Taranto, Nunzio D’Agostino, Monica Rodriguez, Stefano Pavan, Anna P. Minervini, Nicola Pecchioni, Roberto Papa, Pasquale De Vita

**Affiliations:** ^1^Research Centre for Cereal and Industrial Crops (CREA-CI), Foggia, Italy; ^2^Department of Agricultural Sciences, University of Naples Federico II, Portici, Italy; ^3^Department of Agriculture, University of Sassari, Sassari, Italy; ^4^CBV - Interdepartmental Centre for Plant Biodiversity Conservation and Enhancement Sassari University, Alghero, Italy; ^5^Department of Soil, Plant and Food Sciences, University of Bari Aldo Moro, Bari, Italy; ^6^Department of Agricultural, Food and Environmental Sciences, Marche Polytechnic University, Ancona, Italy

**Keywords:** durum wheat, SNP array genotyping, population structure, genetic diversity, private alleles, loci under selection, divergent loci, nitrogen metabolism

## Abstract

The first breeding program in the world for durum wheat was conceived in Italy in the early 1900s. Over the decades, pressure exerted by natural and artificial selection could have progressively reduced the genetic diversity of the durum wheat germplasm. In the present study, a large panel of Italian durum wheat accessions that includes landraces, old and modern cultivars was subjected to genotyping using the Illumina iSelect 15K wheat SNP array. The aim was to assess the impact that selection has in shaping Italian durum wheat genetic diversity and to exploit the patterns of genetic diversity between populations to identify molecular signatures of divergence and selection. Relatively small differences in genetic diversity have been observed among accessions, which have been selected and cultivated in Italy over the past 150 years. Indeed, directional selection combined with that operated by farmers/breeders resulted in the increase of linkage disequilibrium (LD) and in changes of the allelic frequencies in DNA regions that control important agronomic traits. Results from this study also show that major well-known genes and/or QTLs affecting plant height (RHT), earliness (VRN, PPD) and grain quality (GLU, PSY, PSD, LYC, PPO, LOX3) co-localized with outlier SNP loci. Interestingly, many of these SNPs fall in genomic regions where genes involved in nitrogen metabolism are. This finding highlights the key role these genes have played in the transition from landraces to modern cultivars. Finally, our study remarks on the need to fully exploit the genetic diversity of Italian landraces by intense pre-breeding activities aimed at introducing a new source of adaptability and resistance in the genetic background of modern cultivars, to contrast the effect of climate change. The list of divergent loci and loci under selection associated with useful agronomic traits represents an invaluable resource to detect new allelic variants for target genes and for guiding new genomic selection programs in durum wheat.

## Introduction

Durum wheat [*Triticum turgidum* subsp. *durum* (Desf.) Husn] is one of the most important staple crops and it is primarily cultivated in the Mediterranean regions, Southern Europe, and North Africa. Its domestication took place about 10,000 years ago in the Fertile Crescent, as a result of selection and domestication of wild and domesticated emmer (Zohary et al., 2012). As it happened with most crops, the wheat domestication process occurs in four stages (Meyer and Purugganan, 2013; Gaut et al., 2018), during which phenotypic traits changed due to variations in genetic diversity patterns. Natural selection drives adaptive evolution by selecting and increasing the occurrence of beneficial traits contributing to the fitness of individuals that can survive in the wild. At the same time, artificial selection favors traits identified as valuable by the breeders within wild populations, which are characterized by a wide genetic diversity due to demographic factors, including population size and a variation in recombination rate (Jordan et al., 2015).

Differentiation between wild and domesticated populations is affected by artificial (intentional and/or unconscious) and natural selection that contributes to the adaptation of crops to particular climates and diverse environments (McKey et al., 2012).

During the evolutionary history of wheat, the domestication process and the polyploid speciation affected genetic diversity, thus resulting in a reduction of genetic variability, especially in the transition between wild ancestors and landraces (Dubcovsky and Dvorak, 2007; Lopes et al., 2015; He et al., 2019; Maccaferri et al., 2019), which have locally adapted to different Mediterranean environments. A less pronounced genetic bottleneck was observed moving from landraces to modern cultivars under breeding processes (Cavanagh et al., 2013; Maccaferri et al., 2019; Pont et al., 2019), due to the restoration of genetic variability from locally adapted varieties (Gaut et al., 2018).

The wide adaptability of durum wheat to unfavorable climatic conditions and its high commercial value, essentially due to the production of pasta, have promoted numerous breeding programs worldwide. Italy is the leading pasta producing country in the world, with over 20% of the world’s total production (3.4 out of 14.5 million tons in 2014) (Pasta Statistics, 2019)^1^. The first breeding program ever was launched in Italy in the early 1900s and was based on the exploitation of the genetic variability in a few landraces cultivated almost exclusively in Southern Italy, North Africa, and West Asia (D’Amato, 1989; Scarascia Mugnozza, 2005). In fact, most of the varieties released in the early decades of the 1900s were derived from common ancestors, as it is possible to deduce from their pedigree (Laidò et al., 2013). Within this context, SENATORE CAPPELLI (herein after referred to as CAPPELLI), which is assumed to have been selected from the North African landrace JEAN RHETIFAH (sin. JENAH RHETIFAH) and released in 1915 by the renowned breeder Nazareno Strampelli, represents the most popular variety widely used in all breeding programs that were active in Italy until the end of the 1960s (Scarascia Mugnozza, 2005). Its cultivation covered more than 60% of Italy’s durum wheat area for several decades (Bozzini, 1989) and it appears in the pedigree of almost all durum wheat varieties bred in Italy and in many other countries (Laidò et al., 2013).

Early Italian durum wheat breeding programs, exclusively driven by public research institutes, were intended to endow the new varieties with increased resistance to lodging, earliness, improved grain yield, and adaptability to Italian environmental conditions. Afterward, following the introduction of the Law n. 580/67 for Pasta Pureness, which forced factories to produce pasta exclusively with durum wheat, and the Common Agricultural Policy reform, which financially supported the cereal farms in Southern Italy, the cultivation of durum wheat had a further boost coupled with the development of private seed companies. During this time, a new germplasm, mostly from CIMMYT (Mexico), France, and United States was introduced in Italy and the objectives of breeding programs changed. Consequently, modern breeding activities ultimately led to greater uniformity. This means that new varieties could have a lower inclination to adapt to climate change and to tolerate/resist new or re-emerging pests, diseases, and weeds (De Vita and Taranto, 2019; Kahiluoto et al., 2019).

To date, numerous studies have been performed with different molecular markers to analyze how durum wheat genetic diversity varies over time (Maccaferri et al., 2005; Martos et al., 2005; Figliuolo et al., 2007; Laidò et al., 2013; Marzario et al., 2018). Most of these studies have shown a significant decrease in the genetic variability of durum wheat cultivars released in the last century.

Recently, the availability of single nucleotide polymorphism (SNP) array platforms as well as of the wild emmer (Avni et al., 2017) and durum wheat (Maccaferri et al., 2019) reference genomes provide the opportunity to accurately detect changes over time in terms of genetic structure, and localized genomic regions putatively under selection (Manel et al., 2010).

Several studies have investigated genetic variations underlying individual differences in qualitative and quantitative traits associated with the history of durum wheat breeding (Maccaferri et al., 2005; Martos et al., 2005; Figliuolo et al., 2007; Ren et al., 2017; Marzario et al., 2018). However, these studies only used a limited subset of the Italian durum wheat germplasm and considered a short time span compared with the long history of durum wheat breeding. In addition, SNP markers were never used for the genome-wide assessment of the impact of selection on the Italian durum wheat germplasm.

As artificial selection mainly acts on traits of agronomic interest, the identification of loci under selection is more effective if based on the comparison of patterns of genetic variation between populations, rather than on the genotype-phenotype associations (i.e., GWAS). The latter are certainly suitable for identifying highly effective loci and easily measurable traits, but they disregard most of the genetic changes associated with crop improvement (Morrell et al., 2012). Genome-wide analyses based on patterns of linkage disequilibrium (LD) decay, as well as investigations on the genetic differentiation between populations, have already led to the identification of different chromosomal regions under selection in durum wheat (N’Diaye et al., 2018; Maccaferri et al., 2019).

Genetic differentiation between populations can be estimated by the fixation index *F*_ST_ (Wright, 1984), which allows the identification of non-neutrally evolving loci (i.e., loci under selection) between two or more populations from genome-wide SNP data (Pavan et al., 2017). Highly divergent genetic loci between populations have more extreme *F*_ST_ values (>0.25) that might be associated with either natural or artificial selection (Purfield et al., 2017). The search for molecular signatures of selection is essential to understand their functional or adaptive importance and to direct the efforts of genetic improvement on target regions as well as to develop *ad hoc* breeding strategies in an attempt to restore part of the lost genetic variability (Lopez-Cruz et al., 2015).

Within this motivating context, the aims of this work were to (i) assess the genetic diversity in a large collection of durum wheat accessions (over 250) released since the early 1900s by mean of genome-wide high-density SNP array and highly informative gene-associated markers; (ii) to compare the patterns of genetic variation observed in landraces, old cultivars, and modern varieties, define the structure of the population under study, and better understand the history of Italian durum wheat breeding; (iii) to identify molecular signatures of divergence and selection. This work lays the foundation for expanding the genetic base (especially by exploiting the under-explored genetic variability of landraces) and increasing the genetic diversity of future durum wheat cultivars. In addition, it provides a list of genes/loci under selection, some known and some new, associated with useful agronomic traits to be used in marker-assisted breeding or as potential targets for the new plant breeding techniques.

## Materials and Methods

### Plant Material

Two-hundred-and-fifty-nine durum wheat accessions [*Triticum turgidum* subsp. *Durum* (Desf.) Husn] were included in this study. All data on the samples such as geographical origin, *ex situ* collection site, and passport information are listed in Supplementary Data Sheet S1. The collection included indigenous Italian landraces (LR; no. 85), old cultivars (OC; no. 41), and modern cultivars (MC; no. 133). LR and OC, mainly grown in Southern Italy (Sicily, Sardinia and Apulia), were selected starting from a larger panel of ancient accessions available at the Research Centre for Cereal and industrial Crops (CREA-CI), Foggia, Italy. This dataset includes three accessions belonging to *Triticum turgidum* spp. *turanicum* (namely ETRUSCO, KHORASAN CREA and KHORASAN DV), and four durum wheat accessions from Russia (no. 2) and Tunisia (no. 2), widely cultivated in the past in Italy. RUSSELLO and TIMILIA accessions tagged as LR were representative of five and seven different “local populations” collected in 2010 from farmer fields in Sicily by the personnel of the University of Palermo. We defined OC as those accessions developed through a pure line within landraces (indigenous or exotic) or those that were derived from crosses or mutagenesis of cultivars with landraces in their pedigree. We included five accessions of DAUNO III and CAPPELLI collected from different seed banks. Among these, CAPPELLI-MP was considered a pure cultivar, as it is derived from the conservative selection carried out at CREA-CI, the institute in charge for the maintenance of pure seeds according to the DM07/10/2015 No. 20919. The set of modern cultivars included varieties carrying the semi-dwarf *Rht-B1b* allele, released in Italy between 1974 until 2016. In addition, varieties from other countries (e.g., LANGDON, MEXICALI 75, ALTAR 84 and WEST BREAD 881) were included in the MC group as founders of several Italian cultivars. For the purposes of this study, MC were further split up into three groups based on the year of release: MC1 (released before 1990), MC2 (released between 1990 until 2007) and MC3 (released after 2007). All the plant material was regenerated for 3 consecutive years before the present study and single plants for each accession were selected for genotyping.

### Genotyping, Physical Mapping and Quality Control of SNPs

DNA was extracted from leaves by applying the CTAB method (Sharp et al., 1988). Genotyping was performed by Trait Genetics (Gatersleben, DE) using the Illumina^®^ iSelect 15K wheat SNP array, which contains 13,600 highly informative gene-associated SNP markers (Muqaddasi, 2017) and is an optimized and reduced version of the 90K iSELECT SNP-chip described by Wang et al. (2014). The physical positions of all SNPs on Zavitan wild emmer (Avni et al., 2017) chromosomes were obtained by aligning sequences harboring each SNP to the reference genomes by BLAST, retrieving only hits with a full-length alignment. SNPs on un-linked chromosomes were filtered out. Additionally, the genetic and physical positions of all SNPs were assigned based on a consensus map (Maccaferri et al., 2005) and Svevo durum wheat chromosomes (Maccaferri et al., 2019).

SNP quality control was performed using Plink v1.07 (Chang et al., 2015). SNPs with a minor allele frequency (MAF) of <1% and a call rate of >10% were excluded from the downstream analysis. Finally, the dataset was pruned for linkage disequilibrium (*r*^2^ = 0.80) using the SNP and Variation Suite (SVS) software package v.8.4.0 (Golden Helix Inc).

### Genetic Diversity Indices and Population Structure Analysis

High quality SNPs were used to compute summary statistics for each of the five pre-defined populations and to infer the population structure. MEGA v.7 (Kumar et al., 2016) was used to calculate the number of polymorphic (P) and parsimony informative sites (PIS) and singleton variable sites (%), while DnaSP v.5 (Librado and Rozas, 2009) was used to calculate haplotypes (%), and haplotype diversity (Hd).

Genalex v.6.5 (Peakall and Smouse, 2012; GenAlEx, 2018) was used to estimate Nei’s genetic variation index (H), Shannon’s index (I), and the percentage of private alleles.

When comparing gene diversity among LR, OC, and MC the ΔGD parameter was used (Vigouroux et al., 2002). This parameter quantifies the relative deficit of gene diversity (ΔH) between two generic groups of A and B, and it is calculated as 1 – (H_B_/H_A_) when H_A_ > H_B_ and as = (H_A_/H_B_) – 1 when H_A_ < H_B_. The statistics vary between −1 and 1 and is positive when group A has a higher diversity than group B. We calculated this parameter assuming that H_LR_ > H_OC_ > H_MC_.

The analysis of the population structure was evaluated using different methods. Plink v1.07 (Chang et al., 2015) was used to formulate the multidimensional scaling (MDS) plot based on a matrix of genome-wide IBS (identity-by-state) pairwise distances. A neighbor-joining tree was generated using MEGA v.7 (Kumar et al., 2016) and a total of 1,000 replicates were used to generate bootstrap values. FigTree v.1.4.3 (Rambaut, 2009) was used for the graphical visualization of the tree.

Finally, ADMIXTURE version 1.23 (Alexander et al., 2009; Alexander and Lange, 2011) was used to better define the population structure and to assign individuals to different genetic groups using the following parameters: 10-fold cross-validation (CV) for sub-populations (K) ranging from *K* = 1 to 20 and 1,000 bootstrap replicates. CV scores were used to determine the best *K* value. Clustering was performed by setting the number of hypothetical sub-populations (the K parameter) equal to 3 (i.e., LR, OC and MC), 5 (i.e., LR, OC, MC1, MC2, and MC3), and the best value of *K* as indicated by the CV score. We assigned each individual to a specific sub-population when the sub-population membership coefficient (qi) was higher than 0.60. Pairwise genetic distance between sub-populations was estimated using the Weir and Cockerham’s average *F*_ST_, implemented in SVS v.8.4.0.

### Linkage Disequilibrium Analysis

The *r*^2^ statistic was estimated for each pair of SNPs using Plink v1.07 (Chang et al., 2015) to investigate the inter- and intra-chromosomal linkage disequilibrium (LD) decay both within each sub-population (i.e., LR, OC, MC) and the entire population. In case of LR and MC, we considered only 41 accessions (the same number of accessions in the OC sub-population), to exclude possible bias due to the different sizes of sub-populations (Supplementary Data Sheet S1). Additionally, to minimize the sampling effect, we randomly sampled 41 accessions from the LR and MC sub-populations 10 times. On those datasets we performed an ANOVA and applied the Tukey–Kramer test (JMP vs. 7.0, SAS Institute Inc., Cary, NC, United States) to check on possible differences among average *r*^2^ values (within and across chromosomes). The *r*^2^ values of one randomly extracted set of 41 individuals for each of the LR and MC sub-populations were then plotted against the physical distance (in Mb), and the Hill and Weir function was used to describe the decay of *r*^2^ (Marroni et al., 2011). We used the 95th percentile of the inter-chromosomal LD distribution to indicate the r^2^ threshold below which pairwise loci are assumed to be un-linked (Breseghello and Sorrells, 2006). The intersection points between the LD curve and the LD threshold indicates the value of LD decay for each sub-population under study. A value of LD decay estimated using a threshold of *r*^2^ = 0.2 (commonly used) was reported to allow a comparison of the results of the present study with previous ones (e.g., Kidane et al., 2017).

### Identification of Divergent Loci and Signatures of Selection

We evaluated the contribution of divergent selection at single loci (i.e., selection that acts in contrasting directions in two or more populations; Rundle and Nosil, 2005) using the population-based pairwise *F*_ST_ index, as implemented in SVS v. 8.4.0.

The *F*_ST_ at individual SNP loci was estimated using the Weir and Cockerham formula (Weir and Cockerham, 1984) by pairwise comparisons between the sub-populations LR, OC, and MC. The 95% confidence interval around the *F*_ST_ value was calculated using the percentile-t bootstrapping technique (Leviyang and Hamilton, 2011).

Signatures of selection were identified using both BayeScan 1.2 (Foll and Gaggiotti, 2008) and PCAdapt v3.0.2 (Luu et al., 2017). BayeScan was run with 20 pilot runs, 10,000 iterations, a prior odds value of 10, a thinning interval of 10 and a false discovery rate (FDR *q*-value) < 0.05. We performed an outlier test by comparing the LR, OC, and LR sub-populations. In addition, we input a SNP genotype matrix with individuals divided into K sub-populations (i.e., the best value of *K* by ADMIXTURE). It is worth noting that admixed accessions were filtered out to avoid possible biases.

Four distinct PCAdapt runs (i.e., whole population, LR + OC, LR + MC and OC + MC) were computed to identify outlier SNP loci related to artificial selection and cultivar diversification. In order to choose the cut-off for outlier detection, the R package *q-*value v2.5.2 was chosen, using *K* = 20, the Mahalanobis distance method, an additional SNP filtering step (MAF > 0.05) and a *p*-value Bonferroni adjustment at *p* < 0.05, and a false discovery rate (*q-*value) threshold (<0.01). Physical and genetic positions of outlier SNPs were used to associate them with already known QTLs.

## Results

### Genetic Diversity Analysis

Out of 13,006 SNPs spotted onto the 15K Infinium iSelect array, 8,491 were physically mapped on the 14 chromosomes of the wild emmer genome. A total of 7,817 high-quality SNPs (1 SNP per 1,518 kb on average) were retained after filtering (see Materials and Methods) and used for downstream analyses. LD pruning was also applied to remove markers in strong LD, thus reducing the dataset to 3,541 SNPs. Indices of genetic diversity were computed to describe the variability within the 259 durum wheat accessions included in the whole collection and among sub-populations (i.e., LR, OC, MC, MC1, MC2, and MC3) (Table 1).

**TABLE 1:** Summary of genetic diversity and nucleotide variation indices at 3,541 SNP markers, calculated for whole collection, the three main groups *a priori* defined (i.e., LR, OC and MC) as well as the three MC sub-groups (i.e., MC1, MC2 and MC3).

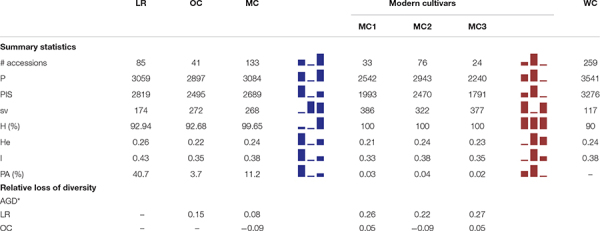	

*Column sparklines showing the trend of each index are also reported. LR, landraces; OC, old cultivars; MC, modern cultivars; WC, whole collection; P, polymorphic sites; PIS, parsimony informative sites; SV, singleton variable sites; H, haplotype; Hd, haplotype diversity; He, Nei’s Index; I, Shannon Index; PA, private alleles. ^∗^He(LR) > He(OC) > He(MC).*

Within the whole collection, 3,541 and 3,276 polymorphic (P) and parsimony informative (PIS) sites were identified, while Nei’s gene diversity (H) and Shannon Index (I) were 0.24 and 0.38, respectively. The number of P and PIS sites slightly decreased moving from LR (*P* = 3,059 and PIS = 2,819) to OC (*P* = 2,897 and PIS = 2,495), while it increased again up to *P* = 3,084 and PIS = 2,689 in MC. Conversely, the trend observed for the number of singleton variable sites was downward moving from LR (174) through OC (272) to MC (268). A higher percentage of private alleles was detected in LR (40.7%) compared with other sub-populations (Table 1 and Supplementary Data Sheets S1, S3). Indeed, landraces revealed a number of private alleles around 11- and 3-folds greater than old and modern cultivars (Table 1 and Supplementary Data Sheets S1, S3). Genetic diversity among the three sub-populations were calculated using Nei’s (H) and Shannon (I) indices. LR show a slightly higher genetic variability (*H* = 0.26, *I* = 0.43) compared with OC (*H* = 0.22, *I* = 0.35) and MC (*H* = 0.24, *I* = 0.38) (Table 1). When the three MC sub-populations are analyzed independently, a slight decrease of diversity was detected in the MC1 and MC3 groups. Gene diversity (ΔH) was positive between LR and OC/MC (ΔH = 0.15 and 0.08, respectively), while a deficit in diversity was observed between OC and MC with ΔH of −0.09 (H_OC_ < H_MC_) (Table 1).

### Population Stratification

The genetic structure of the 259 durum wheat accessions was investigated using parametric and non-parametric approaches to describe the genetic relationships among accessions.

**FIGURE 1:**
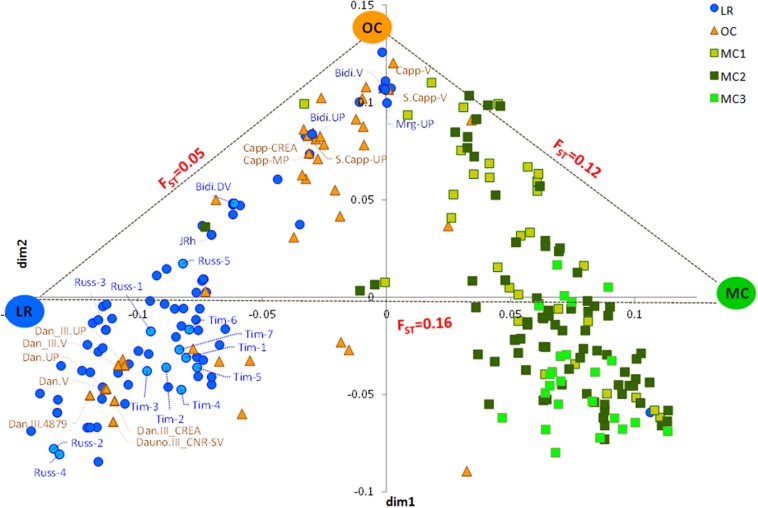
Multidimensional scaling (MDS) plot representing relationships between the 259 durum wheat accessions under investigation. The plot visualizes genome-wide IBS (identity-by-state) pairwise distances between accessions based on 3,541 SNP markers. Colors refer to five different groups (i.e., LR, OC, MC1, MC2, and MC3). Pairwise *F*_ST_ distance values between the three main sub-populations (i.e., LR, OC and MC) were also reported. LR, landraces; OC, old cultivars; MC, modern cultivars; Tim, TIMILIA; Russ, RUSSELLO; S. Capp, SENATORE CAPPELLI; JRh, JEAN RHETIFAH; Mrg, MARGHERITO; Dan, DAUNO.

The MDS plot in Figure 1 shows three main groups. The first dimension (*x*-axis) clearly distinguished LR from MC and, in particular, from MC recently released (MC3), as few MC1 were similar to LR. Most OC were in an intermediate position, although some overlapped with LR and some with MC1. The *y*-axis further distinguished OC from the LR, with some exceptions (Figure 1). Genetic differentiation among the three groups *a priori* defined (i.e., LR, OC and MC) was investigated by computing pairwise *F*_ST_ values, confirming that the genetic differentiation was low between LR and OC (*F*_ST_ = 0.05), moderate between OC and MC (*F*_ST_ = 0.12), and higher between LR and MC (*F*_ST_ = 0.16) (Figure 1).

**FIGURE 2:**
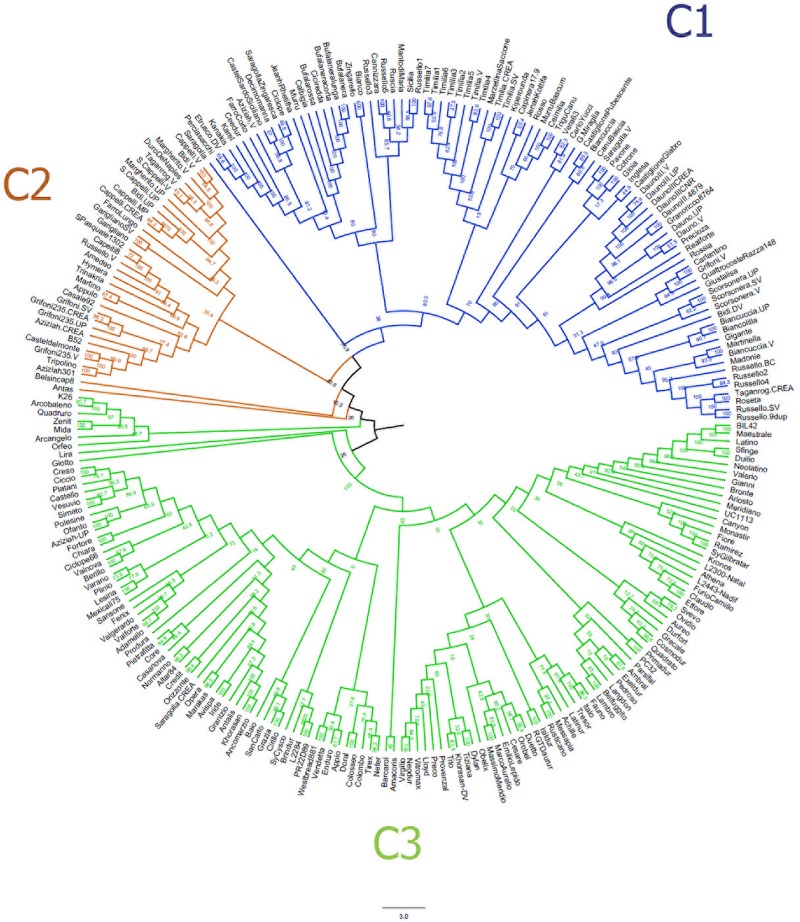
Neighbor-joining tree based on the genetic distances for 259 durum wheat accessions using 3,541 single nucleotide polymorphisms. Accessions are colored in blue (landraces), orange (old cultivars) and green (modern cultivars).

The neighbor-joining clustering (Figure 2) supported the population structure as described by the MDS plot. It shows a clear distinction between LR (C1) and MC (C3), while the majority of OC (C2) clustered between LR and MC. In addition, it provided more accurate information about genetic relationships between accessions.

First, Bayesian clustering was performed by setting the number of hypothetical sub-populations equal to 3 and 5 (see section “Materials and Methods,” Supplementary Data Sheet S1). Then, we considered the value of *K* = 16 as the best number of genetic groups that fit the data (*K* = 16 exhibited the lower cross-validation error compared with other *K* values) (Supplementary Data Sheet S3).

At *K* = 3 (Supplementary Data Sheets S1, S3), two clusters could be clearly distinguished: P1 includes LR and OC, while P3 contains the majority of MC. The P2 cluster combines the remaining MC with a few LRs and OC, with the latter being at the base of the pedigree of accessions in this group. As an example, P2 includes accessions such as APPULO, CAPEITI8, GRIFONI235, HYMERA, BERILLO, and CRESO, all with CAPPELLI as their ancestor (Supplementary Data Sheet S1). Seventeen accessions with admixed ancestry were also identified.

At *K* = 5 (Supplementary Data Sheet S3), the cluster P1 is further divided into two sub-groups: similar to what was observed at K3, P1a includes LR and OC accessions, with the exception of all TIMILIA accessions (LR) that are attributed to the P1b, together with some other OC (GRIFONI 235.V, CASTELDELMONTE, AZIZIAH 301.V, TRIPOLINO, AZIZIAH, B52.V, GRIFONI 235.SV). Similarly, group P2 includes two subgroups: P2a comprises all the LR genetically related to CAPPELLI, while P2b groups all the MC obtained through cross-breeding with CAPPELLI and those mostly released before 1990. The accessions in the cluster P3 are the same as in cluster P3 at *K* = 3, where most of the MC released after 1990 are placed. At *K* = 5, 45 accessions were admixed. Values of pairwise *F*_ST_ distance between the five clusters range from 0.19 (P3 vs. P2b) to 0.37 (P1b vs. P2b) (Supplementary Data Sheet S1).

At *K* = 16 the genetic structure reflects a more composite stratification of the population (Supplementary Data Sheet S3). Specifically, the P1 cluster was divided into six sub-groups (from G1 to G6). G1, G2, G3, and G6 all contain the accessions DAUNO, RUSSELLO, TIMILIA, BUFALA NERA, and their respective relatives. G4 includes the cultivar CEEDUR together with other LRs with which it shares a probable common origin, while G5 consists of two OC, LAMBRO and BELFUGGITO, which have a similar pedigree. The P2 cluster at *K* = 3 was subdivided into four groups (from G7 to G10) and all CAPPELLI accessions were included in G8 with the landraces BIDI and MARGHERITO. The groups G9 and G10 include OC and MC that have CAPPELLI as an ancestor; the accessions in G9 originated from the old cultivar CAPEITI-8, while the accessions in G10 descend from the cultivars VALFORTE and VALNOVA. Lastly, at *K* = 16 the P3 cluster previously identified at *K* = 3 was split into six groups (from G11 to G16), based on pedigree relationships. At this structure level, the number of admixed accessions raised to 96. High genetic differentiation was also observed between groups defined at *K* = 16 (Supplementary Data Sheet S1). The greater *F*_ST_ value (*F*_ST_ > 0.80) was estimated between G5 (BELFUGGITO and LAMBRO, OC) and G6 (BUFALA, LR).

**FIGURE 3:**
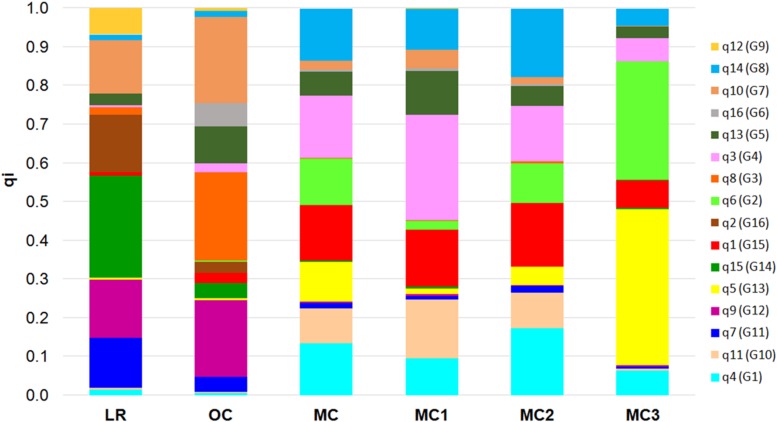
Bar plot that describes the ancestry proportions contributed by K ancestral source populations by calculating the average value of each of the 16 membership coefficients (qi) for each of the six groups *a priori* defined (i.e., LR, OC, MC, MC1, MC2, and MC3). LR, landraces; OC, old cultivars; MC, modern cultivars.

Finally, the 16 membership coefficients (qi) were used to estimate the ancestry proportions for each of the five groups (i.e., LR, OC, MC1, MC2, and MC3), considering qi as the contribution of each K ancestral source population (Figure 3). Results show that not necessarily all the ancestral source populations truly contribute ancestry to the target population: indeed, a variability in the proportion of qi was observed when comparing LR and OC. On the other hand, a clear shift in the ancestry contributions was observed in MC, while a variability in the proportion of qi was recorded following a comparison between the three MC sub-populations. Notably, MC3 includes two membership coefficients with an average value higher than 0.30 (Figure 3). Furthermore, the highest number of private alleles, calculated for each qi, was detected in G2 (Sicilian landraces) and G3 (TIMILIA, LR), while it was the lowest in G5 (LAMBRO and BELFUGGITO, OC) (Supplementary Data Sheet S3).

### Patterns of Linkage Disequilibrium

LD analysis was performed on the three sub-populations following two different approaches. First, all accessions for each population was considered. Then, the number of accessions in LR and MC was reduced to 41 with the aim to numerically balance the three sub-populations.

The Tukey–Kramer test resulted in no significant differences for the average r^2^ values (calculated within and between chromosomes) among the 10 subsets of 41 accessions randomly extracted from LR (*r*^2^ = 0.04, n.s.) and MC (*r*^2^ = 0.11, n.s.) sub-populations. By contrast, significant differences between the values of LD decay of the 10 subsets and the sub-populations that include 85 LR (*r*^2^ = 0.028, *P* < 0.001) and 133 MC (*r*^2^ = 0.038, *P* < 0.001) were observed (Supplementary Data Sheet S1). This indicates that LD decay also depends on the population size (as also observed in previous studies e.g., Bouchet et al., 2012). As a consequence, the values of LD decay among LR, OC, and MC were computed using one subset of 41 accessions randomly sampled from the LR and MC sub-populations. The average values of LD decay of the whole population and the sub-populations were then compared, showing significantly different mean values within and among chromosomes (Supplementary Data Sheet S1).

**FIGURE 4:**
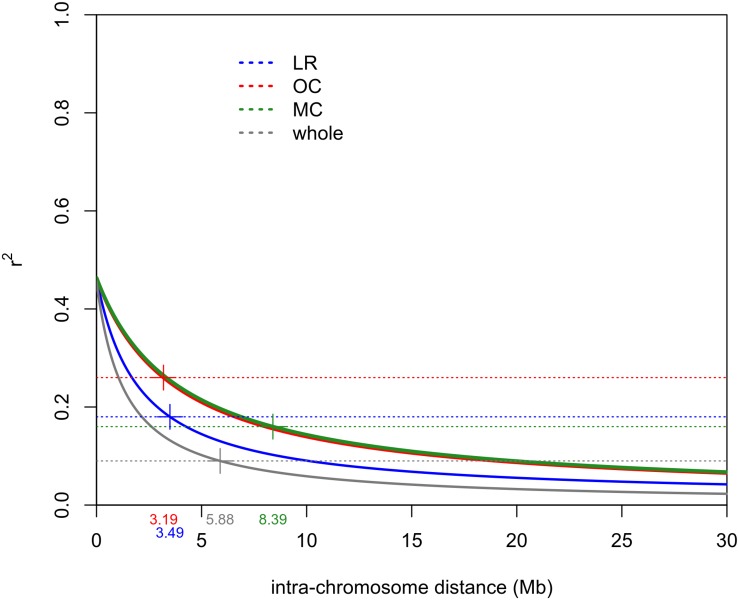
Intra-chromosomal LD decay distance (Mb) evaluated considering only 41 accessions for each sub-population (i.e., LR, OC and MC) and 123 accessions for the whole population. This was done to exclude possible bias due to the different sizes of sub-populations. Dashed lines indicate the *r*^2^ threshold for each dataset. The intersection point between the decay LD curve and the LD threshold was indicated by “+.” LR, landraces; OC, old cultivars; MC, modern cultivars.

The intra-chromosomal LD values were considerably higher for OC (*r*^2^ = 0.078) compared with MC (*r*^2^ = 0.061) and LR (*r*^2^ = 0.056), while the lowest value of intra-chromosomal LD decay was observed within the whole population (*r*^2^ = 0.028) (Supplementary Data Sheet S1). When looking at the LD decay, OC, and LR showed the fastest decay rates (at 3.19 Mb and 3.49 Mb, respectively) (Table 2 and Figure 4), followed by MC (8.39 Mb; Table 2 and Figure 4). When an *r*^2^ value of 0.2 was fixed as the LD decay threshold, the LD extended over a shorter distance for all populations, unless OC (Table 2 and Figure 4).

**TABLE 2:** Per chromosome linkage disequilibrium (LD) decay pattern based on the Hill and Weir function.

	Decay point (Mb)															
	
Populations	1A	1B	2A	2B	3A	3B	4A	4B	5A	5B	6A	6B	7A	7B	Intra chromosomal	LD threshold
**(A)**																
LR (41)	2.91	4.81	2.72	3.03	6.63	3.30	2.71	3.46	4.50	4.55	2.58	3.65	2.84	2.65	3.49	0.18
OC (41)	2.26	4.31	1.44	5.19	5.36	3.11	2.54	2.29	3.60	3.40	3.71	3.24	1.84	2.65	3.19	0.26
MC (41)	7.96	8.09	9.56	14.00	12.34	7.69	3.83	10.35	8.83	13.04	8.41	8.48	5.02	3.01	8.39	0.16
Whole collection (123)	4.66	6.73	5.46	6.31	7.68	5.75	4.00	7.60	6.64	8.49	6.46	5.26	4.22	3.19	5.88	0.09
LR (85)	2.84	4.21	3.63	3.09	8.32	3.60	3.23	5.04	4.71	5.17	3.04	3.76	3.24	2.55	3.80	0.14
MC (133)	7.72	8.37	8.42	12.12	11.28	10.88	5.88	9.44	9.56	14.03	8.48	8.97	5.53	3.87	9.96	0.11
**(B)**																
LR (41)	2.41	4.00	2.25	2.52	5.48	2.73	2.25	2.86	3.72	3.77	2.14	3.02	2.35	2.19	2.90	0.20
OC (41)	3.88	7.39	2.48	8.92	9.20	5.33	4.39	3.95	6.18	5.84	6.36	5.55	3.15	4.54	2.90	0.20
MC (41)	5.41	5.50	6.50	9.52	8.41	5.22	2.60	7.03	6.00	8.87	5.73	5.76	3.42	2.05	5.71	0.20
Whole collection (123)	1.42	2.05	1.67	1.92	2.34	1.75	1.22	2.31	2.03	2.59	1.97	1.60	1.29	0.97	1.80	0.20
LR (85)	1.57	2.34	2.02	1.72	4.63	2.00	1.80	2.80	2.62	2.88	1.69	2.09	1.80	1.42	2.12	0.20
MC (133)	5.41	5.50	6.50	9.52	8.41	5.22	2.60	7.03	6.00	8.87	5.73	5.76	3.42	2.05	3.46	0.20

*LD decay was estimated considering 41 accessions for each sub-population (i.e., LR, OC and MC) and 123 accessions for the whole population. This was done to exclude a possible bias due to the different sizes of the sub-populations. LD decay in LR and MC sub-populations was also estimated considering the total number of accessions of each dataset (i.e., LR = 85 accessions; MC = 133 accessions). The r^2^ decay was calculated based on A) the 95th percentile of the inter-chromosomal LD distribution and B) at fixed *r*^2^ = 0.20. LR, landraces; OC, old cultivars; MC, modern cultivars.*

### Molecular Signature of Divergence and Selection

**FIGURE 5:**
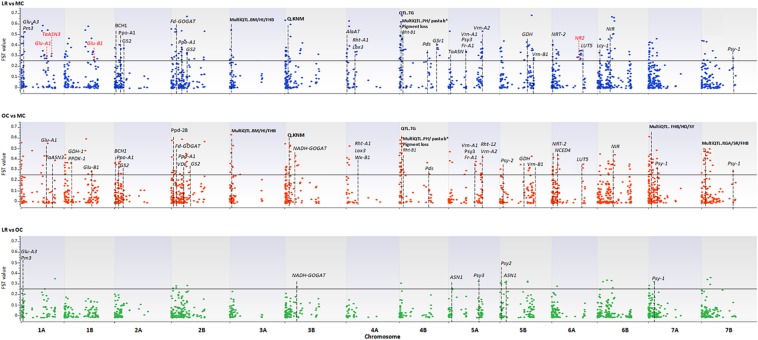
The Manhattan plots show the results of the *F*_ST_ outlier tests performed between pairs of sub-populations (i.e., LR, OC, and MC). Zavitan chromosomes are indicated on the *x*-axis. The *F*_ST_ values (*y*-axis) indicate an outlier SNP when higher than the threshold (*F*_ST_ = 0.25) delineated by a continuous black line. LR, landraces; OC, old cultivars; MC, modern cultivars. Red arrows and the star indicate those genes harboring outlier SNPs. Dashed lines indicate genes/QTLs in the ±6 Mb flanking region of the outlier SNP. Gene name abbreviation: Glu-A1, high molecular weight glutenin subunit; Glu-A3, low molecular weight glutenin subunit; Glu-B1, high molecular weight glutenin subunit; Pm3, powdery mildew resistance; TaASN3, asparagine synthetase 3; NR1, nitrate reductase 1; Fd-GOGAT, ferredoxin-dependent glutamate synthase; NADH-GOGAT, NADH-glutamine oxoglutarate aminotransferase; GDH, glutamate dehydrogenase; GS, glutamine synthetase; Gsr1, Glutamine synthetase 1; NRT-2, nitrate transporter; ASN1/2, glutamine-dependent asparagine synthetase1/2; Rht1/12, plant height; Vrn-1/2, vernalization; Ppd, photoperiod response; Psy, phytoene synthase; Pds, phytoene desaturase; Lyc, lycopene cyclase; Ppo, polyphenol oxidase; Lox3, lipoxygenase; Wx, waxy. QTL name, abbreviation (in bold): BM, biomass; HI, harvester index; FBH, Fusarium head blight resistance; HD, heading date; YR, yellow rust; SR, stem rust; SL, spike length; SLNS, spikelets per spike; TRL, total root length; KNS, kernel number per spike; TW, test weight; KNM, kernel number of main spike; TNR, total root number; ARL, average root length; PRL, primary root length; GY, grain yield; PA, phytic acid; PH, plant height; TKW, thousand kernel weight; days from booting to anthesis (DSB); TG, tough glume and RGA, root growth angle.

A total of 3,541 SNPs were used to detect genomic regions putatively subjected to selection. To identify divergent loci in the three sub-populations (i.e., LR, OC, and MC), we calculated, the pairwise fixation index (*F*_ST_) at individual SNP loci and fixed the significance threshold to >0.25 (Hartl and Clark, 1997). A total of 765 non-redundant outlier SNPs were detected, of which 74, 535 and 467 SNP loci were divergent when comparing LR vs. OC, OC vs. MC, and LR vs. MC, respectively (Supplementary Data Sheet S3) and they are widespread on all 14 chromosomes of the wild emmer genome (Figure 5 and Supplementary Data Sheet S1).

A total of 56 and 83 loci with *F*_ST_ > 0.5 were scored in the LR vs. MC and OC vs. MC, respectively (Supplementary Data Sheet S1). By contrast, the comparison LR vs. OC did not return any divergent SNP locus at *F*_ST_ ≥ 0.5. Only two SNPs (BS00022431_51 and TDURUM_CONTIG11060_433), located, respectively, on chromosomes 4B and 5A, were in common after all three comparisons.

Using the available annotation for the Zavitan genome, four of the outlier SNPs fall into the sequences of high-confidence (HC) genes involved in nitrogen metabolism (no. 2) and quality (no. 2) of wheat. In detail, CAP8_REP_C7343_88 (chr. 1A, *F*_ST_ = 0.27), WSNP_EX_C9343_15514531 (chr. 1A, *F*_ST_ = 0.30), BS00081396_51 (chr. 1B, *F*_ST_ = 0.27), and CAP12_C2701_221 (chr. 6A, *F*_ST_ = 0.25) are located in the coding sequence of the following genes: Glu-A1 (high molecular weight glutenin subunit), TaASN3 (asparagine synthetase 3), Glu-B1 (high molecular weight glutenin subunit), and NR2 (nitrate reductase 2) (Figure 5 and Supplementary Data Sheet S1).

In order to support the identification of divergent loci, we aligned the chromosomal region around individual SNPs with the genomic position of well-known major genes and/or QTLs. Based on the average LD decay calculated on the whole collection, we considered an interval of ±6 Mb around each locus.

One-hundred-and-thirty-four SNPs, which mainly mark the transition from LR/OC to MC, co-localized with genes involved in the nitrogen (N) metabolism (Fd-GOGAT, NADH-GOGAT, GDH, GS, Gsr1, NIR, NRT-2, ASN1), plant height (Rht1 and Rht12), resistance to the powdery mildew fungus (Pm3), vernalization (Vrn-1, Vrn-2), photoperiod response (Ppd), and kernel and semolina color (Psy, Psd, Lyc, Ppo, Lox3, Wx) (Figure 5 and Supplementary Data Sheet S1).

We also investigated possible co-localization of outlier SNP loci (with *F*_ST_ > 0.50) with published QTLs (Maccaferri et al., 2019; Figure 5 and Supplementary Data Sheets S1, S4).

Bayescan analysis did not detect any outlier loci when the three *a priori* defined populations were compared in pairs (i.e., LR, OC, and MC). Then, the analysis was run using exactly 16 sub-populations (16 is the best number of ancestral populations identified by ADMIXTURE). Twenty four outlier SNPs were detected with a *F*_ST_ threshold of 0.18 (FDR *q*-value < 0.05), spanning chromosomes 1B, 2A, 2B, 3A, 3B, 5A, 5B, 6B, 7A, and 7B (Table 3 and Supplementary Data Sheet S3).

**TABLE 3:** List of putative SNP loci under selection (i.e., outlier SNPs) as identified by BayeScan and PCAdapt.

		Zavitan genome				Svevo Genome		
			
SNP ID	SNP Name	Chr	Physical position (bp)	Transcript ID	Annotation	Chr	Physical position (bp)	Genetic position (cM)
***Bayescan***								
IWB5360	BobWhite_rep_c63278_349	1B	524098719	TRIDC1BG049000.5	Ribosomal protein S12/S23 family protein	1B	511074588	n.a.
IWB51641	Tdurum_contig47550_699	1B	571576071	TRIDC7BG068730.6	WD repeat-containing protein 36	7B	682000288	167.4
IWB12175	wsnp_Ku_c26281_36243489	1B	682866630	TRIDC7BG061840.1	F-box family protein	7B	641361493	138.5
IWB12175	wsnp_Ku_c15567_24224486	2A	64105690	TRIDC7BG061840.1	F-box family protein	7B	641361493	138.5
IWB36435	RAC875_c11609_62	2A	70202767	TRIDC6BG070320.2	undescribed protein	n.a.	n.a.	147.5
IWB12175	wsnp_Ex_c59095_60108185	2A	709489657	TRIDC7BG061840.1	F-box family protein	7B	641361493	138.5
IWB25753	IACX5800	2A	735640320	TRIDC6BG021440.13	DNA repair protein RAD5	6B	142207246	64.8
IWB36435	RAC875_c1706_1888	2A	748014324	TRIDC6BG070320.2	undescribed protein	n.a.	n.a.	147.5
IWB10858	Excalibur_c34937_710	2B	4329729	TRIDC5AG037070.16	unknown function	5A	411414173	69.9
IWB6853	BS00035894_51	2B	168932312	TRIDC5AG036430.2	Vacuolar-processing enzyme	5A	405620301	67
IWB62149	Tdurum_contig12836_418	2B	528712939	TRIDC6BG070670.4	NAC domain protein,	6B	685986969	n.a.
IWB57504	Tdurum_contig92604_368	2B	546900973	TRIDC1AG064300.12	Translation initiation factor IF-2	1A	579778835	162.3
IWB57504	tplb0042a21_1091	2B	791585128	TRIDC1AG064300.12	Translation initiation factor IF-2	1A	579778835	162.3
IWB12175	wsnp_Ex_c20250_29303152	3A	692562654	TRIDC7BG061840.1	F-box family protein	7B	641361493	138.5
IWB25753	Excalibur_c878_1249	3B	5531190	TRIDC6BG021440.13	DNA repair protein RAD5	6B	142207246	64.8
IWB57504**	tplb0048g05_866	3B	620410967	TRIDC1AG064300.12	Translation initiation factor IF-2	1A	579778835	162.3
IWB10858	Excalibur_c49550_97	5A	592815919	TRIDC5AG037070.16	unknown function	5A	411414173	69.9
IWB36435**	Ra_c11667_324	5B	572100271	TRIDC6BG070320.2	undescribed protein	n.a.	n.a.	147.5
IWB6853	BS00022886_51	5B	672209240	TRIDC5AG036430.2	Vacuolar-processing enzyme	5A	405620301	67
IWB36435	RAC875_c82589_246	5B	698119978	TRIDC6BG070320.2	undescribed protein	n.a.	n.a.	147.5
IWB62149	Tdurum_contig26001_242	6B	526457265	TRIDC6BG070670.4	NAC domain protein,	6B	685986969	n.a.
IWB10858	BS00085688_51	6B	689707144	TRIDC5AG037070.16	unknown function	5A	411414173	69.9
IWB10858	Excalibur_c18182_464	7A	478920547	TRIDC5AG037070.16	unknown function	5A	411414173	69.9
IWB36435	RAC875_c21489_908	7B	643349812	TRIDC6BG070320.2	undescribed protein	n.a.	n.a.	147.5
***PCAdapt***								
**Whole collection**								
IWB9823	BS00066499_51	2A	3561080	TRIDC2AG001120.1	undescribed protein	chr2A	604345368	124
IWB28807*	Excalibur_c76665_98	2B	684126771	TRIDC2BG071320.5	Protein kinase family protein	chr2B	675427507	137.9
IWA6652*	wsnp_Ku_c18497_27803432	3A	624233333	TRIDC3AG054200.7	unknown function	chr3A	600167153	105.3
IWB74726*	tplb0041o08_752	5A	389585317	TRIDC5AG030720.9	WD-40 repeat family protein	chr5A	385438302	52.9
IWB11373*	BS00084580_51	5A	392615137	TRIDC5AG031100.7	unknown function	chrUn	48373224	54.6
IWB8237*	BS00040623_51	5B	364980353	TRIDC5BG033380.3	Eukaryotic translation initiation factor 3 subunit I	chr5A	390779954	n.a.
IWB33544	GENE-3659_104	6B	164123490	TRIDC6BG023050.2	F-box/kelch-repeat protein OR23	chr6B	153461723	n.a.
IWB3312*	BobWhite_c43557_103	7A	722925739	TRIDC7AG077550.17	DNA-directed RNA polymerases I, II, and III subunit RPABC4	chr7B	716678020	n.a.
IWB64414*	RFL_Contig3607_648	7B	749218177	TRIDC7BG075860.8	Ycf48-like protein	chrUn	153909734	202.9
**LR-OC**								
IWB59770*	RAC875_c6338_2719	1A	351782973	TRIDC1AG029290.6	DnaJ homolog subfamily C member 13	chr1A	343660961	49.7
IWA4852	wsnp_Ex_c8885_14842394	1A	353907087	TRIDC1AG029650.10	Glycerol-3-phosphate acyltransferase, chloroplastic	chr1A	345794081	49.7
IWB40167	Ku_c956_1797	1B	576600580	TRIDC1BG055610.4	Protein BREAST CANCER SUSCEPTIBILITY 1 homolog	chr1B	564019554	87.1
IWB32166	GENE-0968_155	2B	248661787	TRIDC2BG032470.2	receptor kinase 1	chr2B	240557458	91.5
IWB65409	TA001505-1171	2B	250881663	TRIDC2BG032700.8	glycerol-3-phosphate acyltransferase 9	chr2B	242771443	91.5
IWA3742	wsnp_Ex_c40976_47910672	2B	681501339	TRIDC2BG070970.10	SIT4 phosphatase-associated family protein	chr2B	672745921	137.9
IWB60083	RAC875_c67309_634	3A	554642326	TRIDC3AG045420.9	unknown function	chr3A	549634664	83.4
IWA2291	wsnp_Ex_c18223_27035083	3A	599462131	TRIDC3AG051130.6	UDP-glucose 4-epimerase	chr3A	594409411	96.9
IWB8797	BS00060073_51	3B	565414886	TRIDC3BG051640.6	P-loop containing nucleoside triphosphate hydrolases superfamily protein	chr3B	553790145	93.8
IWB74910*	tplb0048g05_866	3B	620410967	TRIDC3BG057580.1	undescribed protein	chr5A	507196211	n.a.
IWB9686	BS00065978_51	3B	646956626	TRIDC3BG059800.8	Lipid A export ATP-binding/permease protein MsbA	chr3B	639467600	n.a.
IWB53771	RAC875_c1377_428	4A	17597227	TRIDC4AG003390.3	Exocyst complex component 6B	chr4A	16826500	18.3
IWB9279	BS00064423_51	4A	618934878	TRIDC4AG052830.1	Protein of unknown function (DUF594)	chr4A	622248523	108.2
IWB13050	CAP11_c7700_247	5B	683750023	TRIDC5BG077490.4	Mitochondrial transcription termination factor family protein	chr5B	672457971	179.4
IWB29394	Excalibur_c92555_283	5B	685236022	TRIDC5BG077700.7	Protein kinase superfamily protein	n.a.	n.a.	179.8
IWB12100*	BS00099879_51	6A	608065330	TRIDC6AG058260.3	C2 domain-containing protein	chr6A	602617850	123.8
**LR-MC**								
IWB7628	BS00026456_51	1A	3273442	TRIDC1AG000400.1	Omega gliadin	chr1A	3851827	2.6
IWB7470*	BS00023201_51	1A	6918899	TRIDC1AG001400.2	Defensin-like protein	chr1A	7558330	6.7
IWB10921	BS00077350_51	1A	11052152	TRIDC1AG002660.1	undescribed protein	chrUn	1292302	n.a.
IWB72042*	Tdurum_contig5008_635	1A	49460159	TRIDC1AG008940.2	Retrotransposon protein, putative, Ty1-copia subclass	chr1A	45805060	37.8
IWB41306	Kukri_c14968_674	1A	165530550	TRIDC1AG018700.5	Myb-related protein 3R-1	chr1A	161596388	35.6
IWB64970	RFL_Contig530_2305	1A	426326790	TRIDC1AG035990.37	MKI67 FHA domain-interacting nucleolar phosphoprotein-like	chr1A	416873382	56.6
IWB60861*	RAC875_c86680_391	1A	467274229	TRIDC1AG040300.5	Subtilisin-like protease SBT3.17	chr1A	456807575	62.8
IWB10042*	BS00067420_51	1A	482795712	TRIDC1AG042170.9	Tetratricopeptide repeat (TPR)-like superfamily protein	chr1A	472637407	70.7
IWA162	wsnp_BE445113A_Ta_2_1	1A	503852292	TRIDC1AG046180.5	Cytochrome P450 superfamily protein	chr1A	493470499	78.9
IWA7573	wsnp_Ra_c11877_19161832	1A	507120245	TRIDC1AG046480.3	undescribed protein	chr1A	496623344	80.2
IWB6743	BS00021728_51	1A	507121068	TRIDC1AG046480.3	undescribed protein	chr1A	496624420	80.2
IWB46717*	Kukri_c58155_786	1A	507262516	TRIDC1AG046540.22	undescribed protein	chr1A	496747426	80.5
IWB57297	RAC875_c37545_289	1A	508119253	TRIDC1AG046740.9	polyubiquitin 10	chr1A	497581205	81.6
IWA6382	wsnp_Ku_c10292_17066821	1A	509682735	TRIDC1AG047040.1	undescribed protein	chr1A	499103571	81.6
IWA6595*	wsnp_Ku_c1642_3232242*	1A	509906809	TRIDC1AG047070.15	protease-related	chr1A	499297726	82
IWA605*	wsnp_BM140362A_Ta_2_2	1A	509907486	TRIDC1AG047070.17	protease-related	chr1A	499298526	82
IWB54285*	RAC875_c16391_426	1A	511262897	TRIDC1AG047240.5	unknown function	chr1A	500702356	82.2
IWB15063*	CAP8_rep_c7343_88	1A	511616947	TRIDC1AG047340.1	Glutenin, high molecular weight subunit 12	chr1B	548462539	83
IWB46448	Kukri_c54678_88	1A	516344130	TRIDC1AG047950.2	H/ACA ribonucleoprotein complex non-core subunit NAF1	chr1A	505711246	84.7
IWB38369*	Ku_c1313_1673	1A	516382360	TRIDC1AG048000.1	NB-ARC domain containing protein	chr1A	505746051	85.1
IWB52379	Ra_c64515_242	1A	516634665	TRIDC1AG048070.16	DNA polymerase nu	chr1A	505968925	85.5
IWA2488	wsnp_Ex_c1997_3757415	1A	516883803	TRIDC1AG048130.6	Disease resistance protein	chr1A	506211205	85.5
IWA2484	wsnp_Ex_c1997_3755945	1A	516885394	TRIDC1AG048130.8	Disease resistance protein	chr1A	506212796	85.5
IWB56727	RAC875_c32452_55	1A	516886283	TRIDC1AG048130.8	Disease resistance protein	chr1A	506213935	86
IWB60627	RAC875_c80876_67	1A	518084931	TRIDC1AG048280.25	Transcription elongation factor Spt5	chr1A	507081023	86.7
IWB29347	Excalibur_c9149_1326	1A	519021913	TRIDC1AG048410.13	Meprin and TRAF (MATH) homology domain-containing protein	chr1A	508070244	87
IWB23140	Excalibur_c1845_4911	1A	519878042	TRIDC1AG048770.42	unknown function	chr1A	508963450	88.8
IWB41872	Kukri_c18017_1696	1A	522867990	TRIDC1AG049220.9	Translation initiation factor IF-2	chr1A	511862997	90.08
IWB34600	IAAV2694	1A	522921603	TRIDC1AG049250.6	TRAM, LAG1 and CLN8 (TLC) lipid-sensing domain containing protein	chr1A	511922847	91.3
IWB54196*	RAC875_c15975_1208	1A	532705756	TRIDC1AG051390.1	undescribed protein	chr1A	525526354	97.4
IWB40255	Kukri_c10121_498	1A	570720073	TRIDC1AG060270.3	unknown function	chr1A	539608929	122.7
IWB9474	BS00065170_51	1A	582636662	TRIDC1AG063120.2	Plant protein of unknown function (DUF247)	chr1A	574962626	142.9
IWB3413	BobWhite_c44947_277	1A	588495700	TRIDC1AG064440.9	Helicase/SANT-associated, DNA binding protein	chr1A	580573915	147.7
IWB47978*	Kukri_c8390_1102	1B	8295417	TRIDC1BG001670.6	Glucan 1,3-beta-glucosidase	chr1B	4925398	15.4
**OC-MC**								
**IWB31732**	GENE-0193_197	1B	373812285	TRIDC1BG033960.12	U1 small nuclear ribonucleoprotein 70 kDa	chr1B	360706881	43
**IWA7520**	wsnp_Ku_rep_c73313_72887199	2B	216214997	TRIDC2BG029650.4	Protein kinase superfamily protein	chr2B	208384507	89.7
IWA6652	wsnp_Ku_c18497_27803432	3A	624233333	TRIDC3AG054200.7	unknown function	chr3A	600167153	105.3
**IWB12651**	CAP11_c1022_117	3A	711466933	TRIDC3AG068260.1	annexin 5	chr3A	705433237	148.5
IWB34975	IAAV5117	4B	503399973	TRIDC4BG042140.3	Heat stress transcription factor A-9	chr4B	501258376	62
IWB74726	tplb0041o08_752	5A	389585317	TRIDC5AG030720.9	WD-40 repeat family protein	chr5A	385438302	52.9
IWB11373	BS00084580_51	5A	392615137	TRIDC5AG031100.7	unknown function	n.a.	n.a.	54.6
IWA3087	wsnp_Ex_c27046_36265198	5A	570069429	TRIDC5AG055200.6	kinase interacting (KIP1-like) family protein	chr5A	536670529	139.4
IWB8237	BS00040623_51	5B	364980353	TRIDC5BG033380.3	Eukaryotic translation initiation factor 3 subunit I	chr5A	390779954	n.a.
**IWA7735**	wsnp_Ra_c2105_4092507	5B	560996162	TRIDC5BG058750.1	undescribed protein	chr5B	552220003	114.9
IWB50957**	Ra_c11667_324	5B	572100271	TRIDC5BG059900.14	polyubiquitin 10	chr5B	562801497	119.3
**IWB43965**	Kukri_c31305_75	7B	433246568	TRIDC7BG037580.4	RING/U-box superfamily protein	chr7B	410570933	76.1

Common outlier SNPs identified by population-based pairwise *F*_*ST*_ and PCAdapt are in bold. Asterisks next to SNP identifiers mark those outlier SNPs detected both by analysis of divergent loci and PCAdapt (*) and among divergent loci, BayeScan, and PCAdapt (**). The underlined SNPs were located in the regions flanking SNPs under selection in Svevo (Maccaferri et al., 2019). The genomic region extending up to 1 Mb proximal to SNP under selection. Chr, chromosome; Un, undefined chromosome; n.a., not available.

The PCAdapt algorithm was run on the whole collection as well as on further three populations (i.e., LR + OC, LR + MC and OC + MC) to detect putative signatures of selection (Table 3), as an alternative approach to detect loci under selection. The PCAdapt on the whole collection tagged nine SNPs as outliers which are located on chromosomes 2A, 2B, 3A, 5A, 5B, 6B, 7A, and 7B. The PCAdapt runs on the LR + OC, OC + MC and LR + MC sub-populations returned 16, 12, and 34 outlier SNPs, respectively (Table 3). Surprisingly, 33 out of 34 SNPs in the latter dataset are in two confined regions on chromosome 1A, while the remaining one is on chromosome 1B. These two regions were investigated further to check for the presence of known genes/QTLs for important disease-resistance and yield-related traits (Supplementary Data Sheets S3, S4).

Interestingly, two loci were outputted by both Bayescan and PCAdapt (Table 3). They were mapped on chromosomes 3B (tplb0048g05_866) and 5B (Ra_c11667_324).

Finally, we combined the results from all three approaches. Two loci were identified by all three methods; 13 outlier SNPs were shared by divergent loci analysis and BayeScan, while 21 SNP loci were outputted by both divergent loci analysis and PCAdapt (Table 3).

## Discussion

It is commonly accepted that the domestication process is associated with a genetic bottleneck that re-patterned genetic variability in wheat (Allaby et al., 2019). Reduction of genetic variability is expected due to the combined effects of drift and selection, but diversity is supposed to be maintained through differentiation among populations (Goldringer et al., 2001). Under natural selection, individuals contribute to the next generation according to their fitness. Basically, both natural and artificial selection determine (very likely to a different extent) the removal of rare alleles, the increase of favorable allele frequencies, the reduction of genetic diversity, and the increase of linkage disequilibrium (Fu, 2015).

During the last century, genetic improvement programs of durum wheat have had a significant impact on productivity gains and the improvement of grain quality as a result of the needs of the processing industry and of consumers’ demands (De Vita et al., 2007; Giunta et al., 2007; Subira et al., 2014). However, pure line selection and the development of varieties with superior agronomic performances has resulted in reduced genetic diversity and increased susceptibility to biotic and abiotic stresses in the improved gene pools (Fu, 2015).

Knowing the molecular targets of artificial selection is essential in guiding the use of the germplasm to breed toward the development of new genetic materials, suitable to meet farmer and market needs while also being able to face climate change (De Vita and Taranto, 2019).

In the present study, a large panel of durum wheat accessions, released for cultivation during the last two centuries in Italy, has allowed us to investigate the impact that selection has had on the genetic structure of the collection under investigation, and to identify and characterize putative molecular signatures of divergence and selection in the durum wheat genome.

A deep knowledge on the extent of genetic diversity in durum wheat is expected to have remarkable importance on the maintenance and use of genetic resources, thus facilitating breeders to achieve profitable diversification in breeding programs.

### Genetic Diversity and Private Alleles Across Populations

The number of Italian durum wheat accessions, which have been investigated in most previous studies (Figliuolo et al., 2007; Laidò et al., 2013; Marzario et al., 2018), was limited to a smaller subset of durum wheat cultivars and/or landraces, and it was restricted to a short time span compared with the long history of durum wheat breeding, thus limiting our understanding of the impact the selection process has had in shaping genetic diversity of the Italian durum wheat germplasm.

To overcome some of these limitations, in this study a high-density SNP genotyping array and a wide representation of Italian durum wheat landraces was also included to counterbalance, from a numerical point of view, the old and modern cultivars developed since the early 1900s. On the one hand, this has allowed us to draw conclusions supported by more data on the effects produced by breeding on genetic diversity, while on the other hand, it has shown how important the exploration of genetic variability in the Italian durum wheat germplasm is, for broadening the genetic base of durum wheat varieties.

Italian landraces were characterized by a moderate level of genetic diversity (He = 0.26), which is comparable with that of Mediterranean (He = 0.24) or Tunisian and Iranian (He = 0.25) landraces, as revealed by the analysis of patterns of genetic diversity using AFLP (Moragues et al., 2007; Nazco et al., 2012) and DArT markers (Fayaz et al., 2018; Robbana et al., 2019), respectively. Relatively small differences in Nei and Shannon indices were observed moving from LR to MC, while we observed that a weak genetic bottleneck (i.e., loss of rare alleles) has been imposed by breeding programs on the old Italian durum wheat (ΔH = −0.09), suggesting that the overall molecular diversity of durum wheat has undergone moderate fluctuations during the 20th century (Maccaferri et al., 2005; Martos et al., 2005; Laidò et al., 2013). This finding is in contrast with what was observed by Marzario et al. (2018) and Figliuolo et al. (2007) who highlighted that the long-term artificial selection process (i.e., breeding programs) has significantly reduced the level of genetic diversity in the durum wheat germplasm. On other hand, several studies on common wheat confirmed that the loss of genetic diversity was not steady during the various decades of the 20th century (Reif et al., 2005; Warburton et al., 2006; van de Wouw et al., 2010).

Significant differences were detected in polymorphic SNPs as well as in private alleles among LR, OC, and MC, with the lowest values in the OC sub-population (Supplementary Data Sheets S1, S3). Data suggest that a fair portion of the genetic diversity was lost in the founders of modern varieties (mostly included in the OC sub-population). Indeed, demographic history (transition from LR to OC) resulted in the selection of few accessions, thus affecting levels of genetic variability (Gaut et al., 2018). An additional aspect is related to the reduction of the genetic heterogeneity of old cultivars due to pure line selection. In this scenario, the spread and intensive cultivation of CAPPELLI and its few closely related varieties for more than two decades was an example of how the genetic basis of mostly cultivated Italian durum wheat cultivars has been narrowed (Bozzini et al., 1988).

The sharp decline in the number of private alleles moving from LR to OC, and the moderate restoration of variability in MC2 is partially in agreement with what has been observed by Ren et al. (2013), who analyzed a worldwide germplasm collection of durum wheat and observed a loss of genetic diversity moving from LR/OC to MC released during the early Green Revolution as well as an upward leap in genetic diversity for those cultivars released during the post-Green Revolution.

The fluctuation of the number of polymorphic SNPs and private alleles was emphasized by a clear shift in the allele frequency spectrum moving from LR to MC (Figure 3), as previously detected by Laidò et al. (2013). Indeed, the changes in genetic diversity during the transition from LR, through OC, to MC seem to be more qualitative than quantitative, thus implying the effect of positive selection and selective sweep in MC2 (Table 1). Changes in the ancestry proportions (i.e., allele frequencies) moving from LR/OC to MC could be attributed to the introgression of new alleles from CIMMYT, France and United States germplasm and/or to the use of mutation-breeding strategies, which have been applied in Italy since the 1960s.

### Population Structure of the Italian Durum Wheat Germplasm

The genetic structure of the collection under study was investigated using three approaches (Figures 1–3 and Supplementary Data Sheet S3). All methods returned convergent results: overall population structure reflects the history of Italian durum wheat breeding that spans from the selection of indigenous or exotic landraces to the development of modern elite varieties.

Most Italian landraces were indigenous, cultivated in specific areas of Southern Italy (mainly in Sicily and Sardinia and to a lesser extent in Apulia, Campania, and Basilicata). They were the results of natural selection over a long period of time and/or of unconscious selection by farmers (Azeez et al., 2018). The continue exchange of seeds among farmers and the introduction of exotic LR from North Africa and West Asia contributed to the maintenance of a large genetic base within local varieties (see Figure 3).

In the past, durum wheats partly flowed from North Africa and Greece through Sicily and other regions of Southern Italy (Ciferri and Bonvicini, 1959). Many of them have been broadly cultivated and occasional natural crosses might have contributed to the increase of the variability of local populations. Two of these local populations in particular, namely RUSSELLO and TIMILIA, have been cultivated over time, especially in Sicily, because of their adaptability to extreme Mediterranean environments (Boggini et al., 1990). In the present study, using the Bayesian approach (*K* = 16), LR grouped into five clusters, two of which include the Sicilian landraces RUSSELLO and TIMILIA (G2 and G3 in Supplementary Data Sheet S3). All RUSSELLO accessions grouped in a single cluster together with other Sicilian accessions. By contrast, TIMILIA accessions were in a separate cluster together with MARZELLINA, a spring sowing durum wheat landrace grown in Campania and Apulia. High genetic distance between accessions of TIMILIA with most of the germplasm analyzed in this study was clearly observed by private alleles, multidimensional scaling, non-parametric clustering, and *F*_ST_ distance analysis (Supplementary Data Sheets S1, S3). This suggests that the genetic base of those accessions is unique, and it is probably associated with the peculiar morpho-phenological and grain quality traits (Muccilli et al., 2011; Taranto et al., 2012; Giancaspro et al., 2016). As a consequence, TIMILIA accessions could be exploited for the introgression of useful alleles in modern cultivars, as they were used to a limited extent, unlike RUSSELLO and other landraces (Boggini et al., 1990).

The transition from LR to OC was characterized by the introduction of new founders, derived from landraces subjected to a selection process. At the beginning of the twentieth century, Nazareno Strampelli, exploiting the genetic variability of landraces of different origin, released the DAUNO III and immediately after the CAPPELLI variety. Our results support the hypothesis that DAUNO III could derive from landraces of Italian origin (De Cillis, 1942), as it grouped with Sicilian and Sardinian landraces.

Moreover, our results (Figures 1, 2) highlight the key role that the old cultivar CAPPELLI played, until 1974, in the selection of new varieties with the semi-dwarf phenotype, but also in the development of modern cultivars released in Italy before 1990. Indeed, CAPPELLI could be considered as a main “ancestor” of the durum wheat germplasm (Autrique et al., 1996).

In the decades following the introduction of CAPPELLI, our results highlight how the repeated use of a few founders caused a reduction in genetic variability of the derived varieties. The latter, in fact, fall in the same cluster or in neighboring clusters (i.e., OC and MC1).

In this work, five accessions of CAPPELLI were collected from different seed banks in order to estimate the possible genetic distance among them and from the accession CAPPELLI-MP, the one present in the Italian National Variety Register. All the CAPPELLI accessions were in the same group (Figures 1, 2 and Supplementary Data Sheet S1), thus suggesting their common origin. Cluster C2 in Figure 2, in particular, also includes the Sicilian landraces MARGHERITO and BIDI and two accessions (DURO DE NAPLES and TAGANROG) of unknown origin. Unexpectedly, the landrace JEANH RHETIFHA was genetically distant from the CAPPELLI group. This result agrees with what was assumed by De Cillis (1927) and has recently been confirmed by Marzario et al. (2018) and Fiore et al. (2019). In addition, given the growing market demand for mono-varietal products and the possibility of re-covering obsolete durum wheat varieties as “conservation varieties,” the obvious genetic similarity between BID\`I, MARGHERITO and CAPPELLI raises the need of developing new traceability tools to mitigate possible food fraud risks.

### Linkage Disequilibrium

Several studies estimated the extent of LD and its decay (genetic and physical distances) in durum wheat using different classes of molecular markers (Laidò et al., 2013; Maccaferri et al., 2015; Roselló et al., 2019). The availability of a high-density SNP map (Wang et al., 2014) and the reference genome sequence of wild emmer and durum wheat (Avni et al., 2017; Maccaferri et al., 2019) has made the LD estimate more precise than in the past, despite the persistent complexity of the wheat genome, allowing one to estimate the physical distance (Mb). The variation of LD patterns across the three sub-populations (i.e., LR, OC and MC) reflects what has been discussed so far in the literature (Laidò et al., 2013; Maccaferri et al., 2015; Roselló et al., 2019). At *r*^2^ = 0.2, the LD decay distance for LR and MC is comparable with that reported by Maccaferri et al. (2019) (Table 2) and it is less than the value (51.3 Mb for *r*^2^ < 0.2) reported by Bassi et al. (2019). Maccaferri et al. (2019), reported LD decay values (*r*^2^ = 0.2) at 1.6 and 4.5 Mb for LR and MC, respectively. The increase of the LD decay distance in MC could be due to selective pressure on genomic loci. Altogether, these data suggest that selection by breeders has resulted in the conservation of haplotype blocks harboring beneficial gene combinations. The LD decay distance also varies across chromosomes. Even if there are few works in which LD decay have been reported in physical distance, it is possible to compare our results with previous LD patterns evaluated in cM. Among the MC chromosomes, 1B, 4B, and 6A showed the slowest decay both as physical and genetic distances. These results were partially confirmed in durum and bread wheat (Somers et al., 2007; Voss-Fels and Snowdon, 2016; Kidane et al., 2017; Rufo et al., 2019).

### Outlier Loci and Selection Signatures

In this work, we identified divergent loci between landraces and old and modern cultivars, and detected signatures of divergence and putative genes under selection using three different methods that returned a number of outlier SNPs that decrease moving from *F*_ST_ analysis (765 non-redundant SNPs with *F*_ST_ > 0.25), through PCAdapt (67 non-redundant SNPs), to Bayescan (24 non-redundant SNPs), of which only two were identified by all three methods (see Table 3).

About 94% of SNPs (718 out of 765 outliers) were in hot spot regions in which genes/QTLs for different traits have been previously reported in bread (Cavanagh et al., 2013) and durum wheat (Maccaferri et al., 2019). A larger number of SNPs were detected comparing LR vs. MC, and OC vs. MC (Supplementary Data Sheets S1, S3). In fact, at *F*_ST_ values > 0.25 populations might differ due to divergent selection, artificial selection, genetic drift, and non-random mating (Fu, 2015).

By looking at the SNPs with *F*_ST_ > 0.25, most were in genomic regions well known to influence plant traits, such as height, photoperiod response, vernalization requirements, and frost tolerance (Figure 5). This result was consistent with the main objectives of breeding programs since MC are more productive with a higher harvest index (HI) (De Vita et al., 2007; Royo et al., 2008; Fayaz et al., 2013), less photoperiodic sensitivity, and fewer vernalization requirements (Giunta et al., 2007) and, with better overall end-use quality traits than LR (De Vita et al., 2007; Nazco et al., 2012). This particularly emerges when LR/OC are compared with MC, probably because plant height and flowering time represent critical traits that facilitate harvesting and adaptability of new varieties to extreme environments in Southern Italy (De Vita et al., 2010).

In addition, numerous studies have highlighted the key role of several nitrogen metabolism-related genes in the domestication process of various crop species, such as maize (Hufford et al., 2012), sunflower (Chapman et al., 2008), common bean (Bellucci et al., 2014), sorghum (Massel et al., 2016), and wheat (Gioia et al., 2015; Beleggia et al., 2016).

Our findings revealed several N use efficiency (NUE)-associated genes (responsible for the uptake, assimilation and remobilization of nitrogen in the grain) and remarked the key role these genes have had in the transition from LR to MC (Garnett et al., 2009). Increased above-ground biomass, seed production, grain protein, and yield usually results in a corresponding increase of NUE in crops (Masclaux-Daubresse et al., 2010). Varieties with improved NUE, however, were indirectly selected by breeders as a result of choosing higher yielding varieties (Cormier et al., 2013).

Several genes of primary N uptake and assimilation have been identified as potential candidates to enhance NUE in crops *via* genetic engineering. Our data indicate high-affinity nitrate transporter 2 (NRT2), nitrate reductase (NR), and Nitrite reductase (NiR) on chromosome 6A and 6B (Figure 5). NRT2 is involved in nitrate uptake and it is regulated by demand-supply of N (Li et al., 2019). NR first reduces the nitrate into nitrite in cytosol followed by reduction to ammonium in plastid. NiR catalyzes the reduction of nitrite to ammonium in the second step of the nitrate assimilation cycle (Boisson et al., 2005).

Assimilation of ammonium depends on glutamine synthetase (GS), the enzyme responsible for converting inorganic N into the organic amino acid glutamine. GS has been one of the most extensively studied enzymes and has been used to improve NUE in wheat (Thomsen et al., 2014). Glutamine is, in turn, converted into two glutamate molecules by the glutamine oxoglutarate aminotransferase (GOGAT) enzyme with its reducing power that varies depending on the isoform.

Further two genes, NADH-GOGAT and Fd-GOGAT, located, respectively, on chromosomes 3B and 2B were identified as loci under selection. NADH-GOGAT has long been considered one of the major candidate genes for cereal NUE, as described by Quraishi et al. (2011) and Nigro et al. (2019). In addition, the QTL harboring NADH-GOGAT overlaps with those controlling seedling root traits, thus suggesting how important the contribution of morphological root traits to NUE is (Li et al., 2015).

Also, glutamate dehydrogenase (GDH) plays a key role in the maintenance of N-C balance by liberating ammonium during senescence (Labboun et al., 2009). Additional enzymes, namely ASN1, ASN2, and ASN3, that belong to the cytosolic asparagine synthetase (AS) family, also participate in ammonium assimilation (Lam et al., 2003). Asparagine has long been considered an important nitrogen transport and storage molecule, in particular during the grain filling period (Lea et al., 2007; Masclaux-Daubresse et al., 2010). Considering this, QTL is also associated with the texture of the glumes on chromosome 4B (Supplementary Data Sheet S1), as it is responsible for remobilization and accumulation of nitrogen in seeds, as demonstrated by Kohl et al. (2012, 2015) in barley. Recent studies showed that ASN gene family members also act in response to nitrogen levels and other abiotic stresses (Curci et al., 2018). Presently, the interest in the mechanisms underlying asparagine synthesis, break-down, and accumulation in crops has increased further, as asparagine is the precursor of acrylamide, a chemical hazard in the food chain (Rapp et al., 2018).

The over-expression of the NADH-GOGAT gene causes an increase in grain yield in both wheat and rice due to the increase in kernel weight; its silencing, instead, is associated with weight reduction of the kernel and a consequent decrease in yield per plant (Yamaya et al., 2002; Lu and Hu, 2011). This points to the essential role NADH-GOGAT plays in nitrogen utilization and grain filling. The importance of N metabolism-related genes on grain yield was also corroborated by studies conducted on maize that showed that the activity of NR and/or NiR enzymes as well as the level of the GS-1 isoform in leaves impact on plant growth (Masclaux et al., 2001). The introduction of an extra copy of the GS gene in wheat improved the glutamine content in leaves 4-fold, resulting in an increased grain number and N content in the grain (Habash et al., 2001).

A comparison between old and modern cultivars in bread and durum wheat grown at multiple N rates, indicated a better response to N for gains in grain yields in the modern varieties, which was reflected in increased uptakes and NUE (Ortiz-Monasterio et al., 1997; Brancourt-Hulmel et al., 2003; Guarda et al., 2004; Giambalvo et al., 2010; Cormier et al., 2013; Gioia et al., 2015). This indicates that MC are characterized by a higher responsiveness to improved environmental conditions. In this scenario, the new durum wheat breeding programs should be considered as suggested by Curci et al. (2017), Beleggia et al. (2016), and Saia et al. (2019). Indeed, many genes involved in N absorption and assimilation were differentially expressed under N-stress, compared with the normal condition. In particular, comparing emmer and durum wheat cultivars, N-metabolism genes were up-regulated rapidly after N treatment emphasizing the key role played by these genes in plant growth and development.

Finally, since cooking properties of pasta are also related to high content of N in the grain, it could be very interesting to modulate the accumulation of proteins in durum wheat grains as recently suggested by Nigro et al. (2019). Grain protein content, together with gluten strength and the yellow color of grain and semolina, are indeed desirable features of high quality durum wheat (Troccoli et al., 2000).

The concentration of yellow pigments is mandatory to achieve the bright yellow color seen in pasta products, a consumer’s requisite, thus becoming an important target in modern breeding programs (Digesù et al., 2009). Our data revealed that Psy genes (Psy1, Psy2, Psy3) were under selection in modern cultivars. Two of them, located on chromosome 5 (Psy2) and 7 (Psy1), are involved in carotenoid biosynthesis (Pozniak et al., 2007). The yellow color of pasta is not only determined by the accumulation of carotenoids in the grain but is also due to the action of LOX, peroxidase, and PPO enzymes, which play a role in reducing or “bleaching” flour pigments during pasta making (Borrelli et al., 1999). Among the outlier SNPs mapped on chromosomes 2A, 2B, and 4A, Ppo-2A, Ppo-2B, and Lox3 genes were associated in LR/OC to MC transition (Taranto et al., 2015), thus confirming that MC have significantly lower PPO activity than LR/OC (Taranto et al., 2012).

Bayescan and PCAdapt were used to detect putative loci under selection. The smaller number of Bayescan outliers was expected as the two methods differ in type I (false positives) and type II (false negatives) error rates (Narum and Hess, 2011; Luu et al., 2017). Only two SNPs were in common and were located on chromosome 3B and 5B, respectively (Table 3). The flanking regions of two SNP markers were evaluated according to LD decay distance that is about 5Mb for chromosomes 3B and 8 Mb for chromosome 5B (Table 2). The region on chromosome 3B harbors many genes responsible for the response to abiotic and biotic stresses, such as TBC1, RBOH (respiratory burst oxidase protein F), Pectate lyase, and Zinc finger protein CONSTANS-LIKE 5 (Atif et al., 2019; Lai et al., 2020; Saijo and Loo, 2020), genes involved in the nitrogen assimilation process (Ureidoglycolate hydrolase) (Muñoz et al., 2006) and in the regulation of auxin polar transport (Interactor of constitutive active ROPs 1). Candidate genes putative under selection on chromosome 5B are well known to be involved in different biological processes in wheat. Notably, we found auxin/indoleacetic acid responsive protein, lipoxygenase 1, strictosidine synthase, phototropic-responsive NPH3, chalcone synthase, phytochrome C, ethylene response factors, and tornado 1 genes involved in plant growth, grain development and response to biotic and abiotic stresses (Singla et al., 2006; Feng et al., 2012; Pielot et al., 2015; Phukan et al., 2017; Zou et al., 2017). This findings could represent an important starting point for the identification of genes involved in plant adaptation and to describe better durum wheat evolution.

Finally, although PCAdapt results were not confirmed by Bayescan, loci on chromosome 1A certainly deserve a focus (Supplementary Data Sheet S3). Indeed, as for the properties of gluten the genomic regions in the range from 2.6 to 6.7 cM, and from 80 to 83 cM on chromosome 1A, are well known for harboring gliadin and glutenin loci and several QTLs, meta-QTLs, and candidate genes that influence agronomically important traits (Supplementary Data Sheets S3, S4). The selective pressure on these genes/QTLs marked the transition from LR, through OC, to MC. In particular, the locus Glu-1 was reported to be nearly fixed in modern bread (Joukhadar et al., 2019) and the durum wheat germplasm (Maccaferri et al., 2019).

## Conclusion

Relatively small differences in overall genetic diversity have been observed in the panel of durum wheat accessions including landraces, and old and new varieties selected and cultivated in Italy in the last 150 years. Based on our results, the increase of LD decay and the shift in the allele frequency spectrum were the main effects produced by the demographic history along with the selection by farmers. Our study also remarked how under-exploited the genetic variability that characterizes the Italian durum wheat landraces is, and the need to recover the untapped variability present in the landraces. Finally, the list of loci under selection highlighted the key role NUE genes have had and could have in the near future to improve quality traits and agronomic performances of novel durum wheat varieties. Nowadays, it is necessary to develop new high yielding varieties that are able to grow with reduced agro-chemical inputs and under fluctuating climatic conditions (De Vita and Taranto, 2019). Sustainable agriculture requires varieties with improved traits such as weed suppression ability, enhancement of nutritional value, higher NUE under low and high N conditions, and optimization of plant interactions with microbial communities in the soil (see Brummer et al., 2011). To meet all those challenges, breeding strategies must necessarily evolve to exploit genetic resources more thoroughly and must prefer interdisciplinary approaches capable of improving their efficiency and effectiveness (Taranto et al., 2018).

## Data Availability Statement

Supplementary Data Sheet S2 collects the command lines used for running ADMIXTURE and Bayescan and the R script for PCAdapt. The filtered VCF file has been uploaded to the Figshare repository (doi: 10.6084/m9.figshare.11835663).

## Author Contributions

FT and PD designed the experiment. FT, PD, and RP established the durum wheat collection. ND’A carried out part of the bioinformatic analysis. FT, ND’A, and SP performed the genetic diversity analyses. MR performed the Linkage Disequilibrium analysis. FT, ND’A, and AP detected loci under selection. FT, ND’A, MR, SP, NP, RP, and PD were involved in the data interpretation. FT, ND’A, and PD wrote the manuscript. All authors critically revised the manuscript. All authors read and approved the final manuscript.

## Conflict of Interest

The authors declare that the research was conducted in the absence of any commercial or financial relationships that could be construed as a potential conflict of interest.

## Footnotes

1 http://www.internationalpasta.org/index.aspx?id=7
