# Supplementary material for: Whole Genome Scan Reveals Molecular Signatures of Divergence and Selection Related to Important Traits in Durum Wheat Germplasm
Source: Front Genet. 2020 Apr 21;11:217. doi: 10.3389/fgene.2020.00217 (PMC7187681; doi:10.3389/fgene.2020.00217)
Supplement: Supplementary file 2 [file Data_Sheet_2.docx]

**# command line for running ADMIXTURE**

for K in 1 2 3 4 5 6 7 8 9 10 11 12 13 14 15 16 17 18 19 20; do /opt/admixture_linux-1.3.0/admixture --cv=10 -B1000 -s 500 -c 5 -j16 /path/to/input/file $K | tee log${K}.out; done

**# command line for running Bayescan**

/opt/BayeScan2.1/binaries/BayeScan2.1_linux64bits /path/to/SNP/genotypes/matrix -nbp 20 -pr_odds 10 -thin 10 -n 10000 -od /path/to/output/dir/

# **Running PCAdapt**

library(pcadapt)

library(qvalue)

file.choose()

"/path/to/input/file"

setwd("/path/to/working/dir")

filename <- read.pcadapt("input_file", type = "ped")

x <- pcadapt(input = filename, K = 20)

pdf("screeplot.pdf")

plot(x, option ="screeplot")

dev.off()

y <- pcadapt(filename, K = 16, method = "mahalanobis", min.maf = 0.05, ploidy = 2, LD.clumping = NULL, pca.only = FALSE)summary(y)

pdf("scores12.pdf")

plot(y, option = "scores")

dev.off()

pdf("scores34.pdf")

plot(y, option = "scores", i = 3, j = 4)

dev.off()

qval <- qvalue(y$pvalues)$qvalues

alpha <- 0.01

outliers <- which(qval < alpha)

length(outliers)

padj <- p.adjust(y$pvalues,method="bonferroni")

alpha <- 0.05

outliers <- which(padj < alpha)

length(outliers)

print(outliers)
