# Supplementary material for: Whole Genome Scan Reveals Molecular Signatures of Divergence and Selection Related to Important Traits in Durum Wheat Germplasm
Source: Front Genet. 2020 Apr 21;11:217. doi: 10.3389/fgene.2020.00217 (PMC7187681; doi:10.3389/fgene.2020.00217)
Supplement: Supplementary file 3 [file Data_Sheet_3.docx]

Supplementary Figures


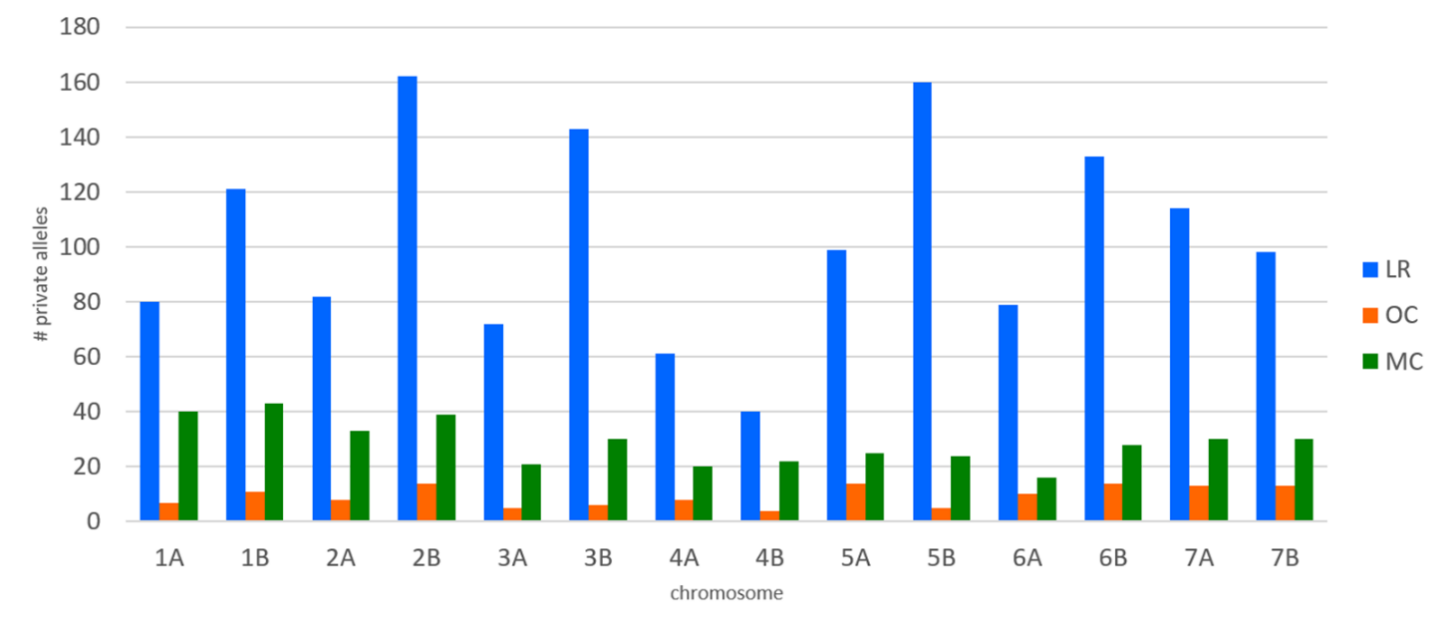
**Supplementary Figure** 1**.** Distribution of private alleles across the wild emmer chromosomes in the three sub-populations. LR= landraces; OC= old cultivars; MC= modern cultivars

**Supplementary Figure 2.** Evaluation of the best grouping number (K) for the 259 durum wheat accessions. Plot shows the cross-validation (CV) error for K values ranging from 1 to 20. The red circle indicates the best value of K that corresponds to lowest value of the CV error. This specifies that the 259 durum wheat accessions are likely subdivided into 16 genetic groups.


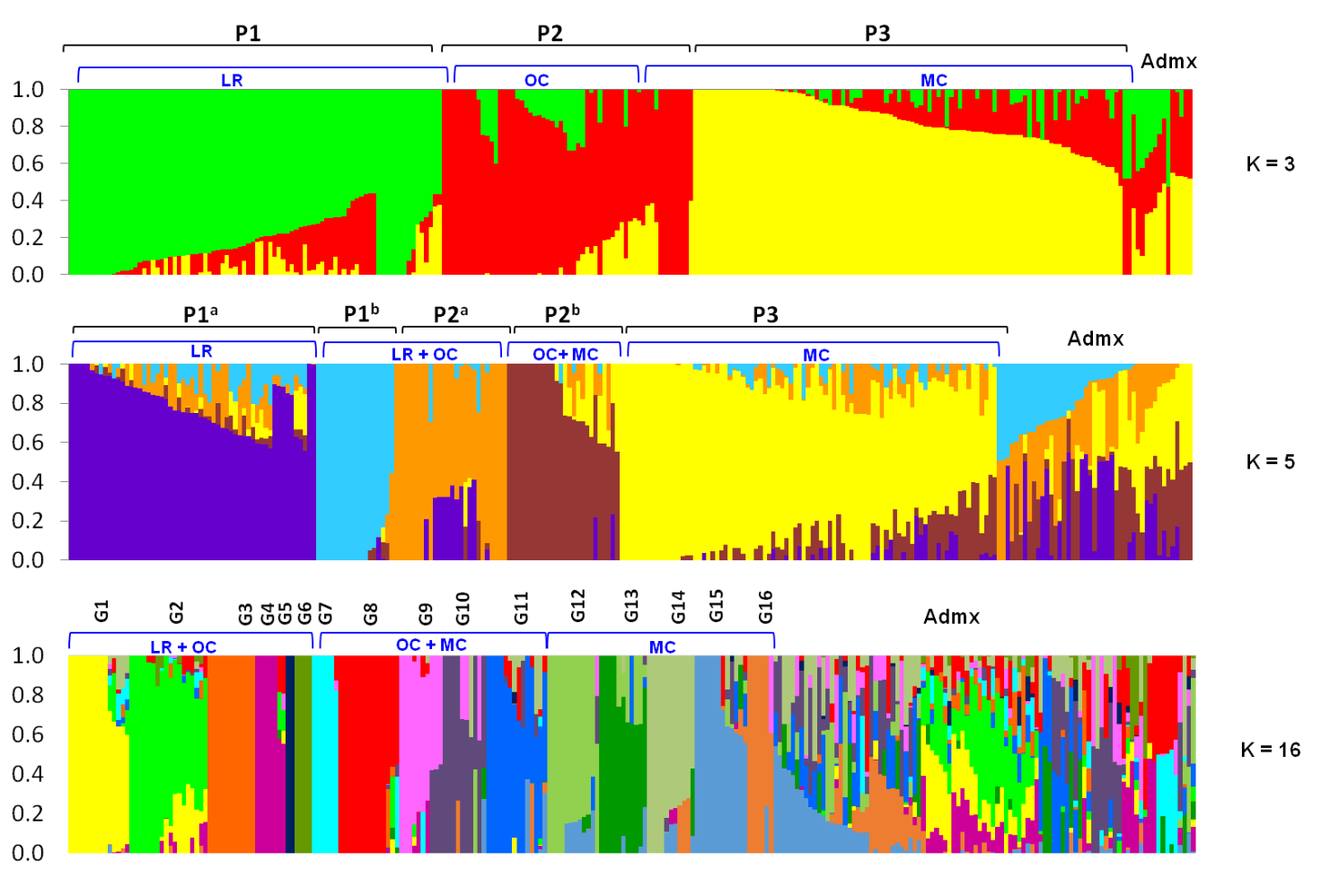


**Supplementary Figure 3.** Population structure of 259 durum wheat accessions derived form 3,541 SNPs. Model-based admixture bar-plots in the hypothesis of 3, 5 and 16 clusters (K). Each accession is represented by a thin vertical bar, which is in turn partitioned into K coloured segments whose length is proportional to the estimated membership coefficient (qi). For each accession, group membership for different values of K is given in Table S1.

LR= landraces; OC= old cultivars; MC= modern cultivars.

**Supplementary Figure 4.** Distribution of private alleles calculated for each membership coefficient (qi) generated at K = 16 by ADMIXTURE. The correspondence between qi and groups (K) was in Table S1.


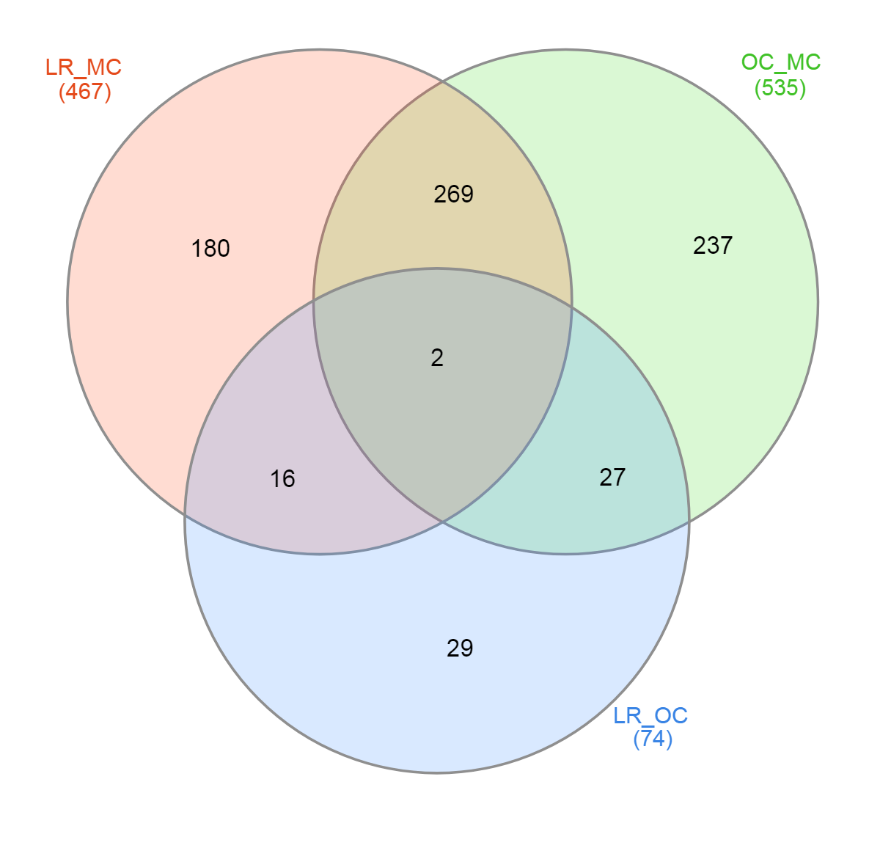


**Supplementary Figure 5.** Venn diagram describing the overlap of divergent SNPs identified by comparing the sub-populations LR, OC and MC and using the population-based pairwise fixation index (F_ST_). The number of SNP exceeding the significant threshold of 0.25 is shown.

LR= landraces; OC= old cultivars; MC= modern cultivars.


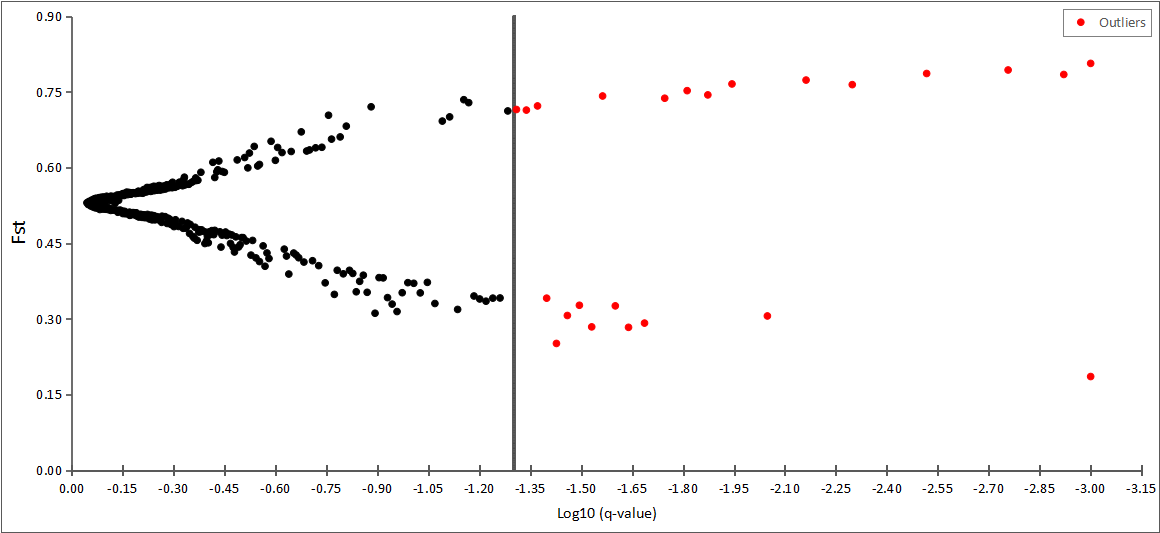


**Supplementary Figure 6.** Outlier SNPs identified by BayeScan follwing the comparison of 16 ancestral populations of durum wheat unless admixed accessions. BayeScan indicates evidence for selection (the vertical line corresponds to log10 (BF) > 1.31) for twenty-four SNPs (in red). Log 10(q value) are on the x-axis and F_ST_-values on the y axis.


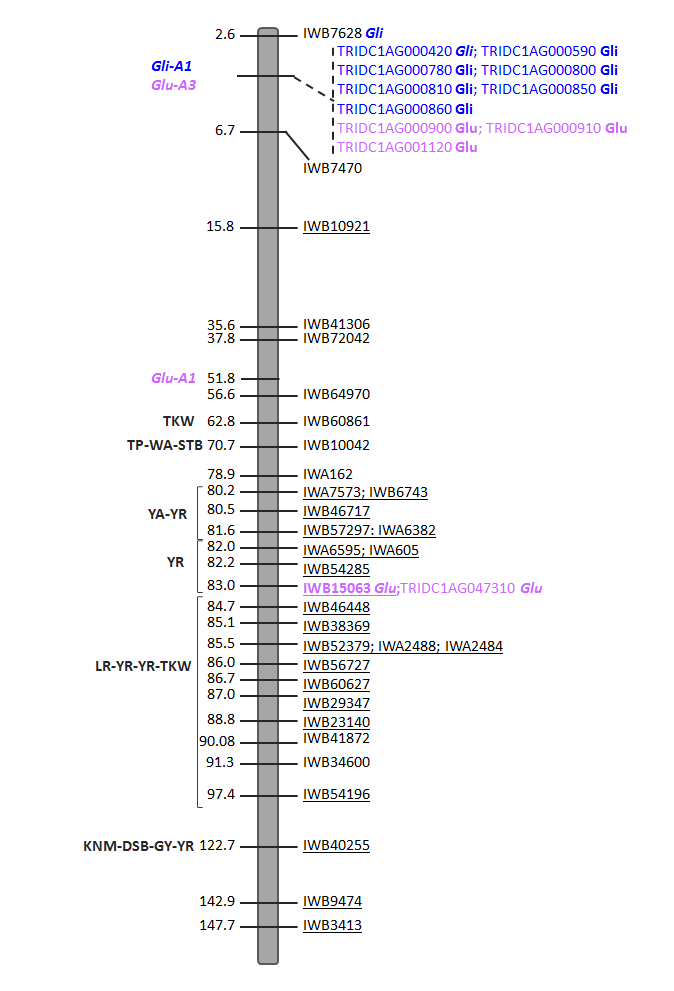


**Supplementary Figure 7.** Ideogram of the chromosome 1A that shows the list of outlier SNPs detected by PCAdapt (run LR+MC) associated with the QTL/gene list based on a priori knowledge of their functions. Outlier SNPs are underlined. Gliadin (*Gli-A1*) and glutenin (*Glu-A3* and *Glu-A1*) transcripts are indicated in blue and violet, respectively. Known QTLs are listed on the left of the chromosome 1A. QTL name, abbreviation: thousand kernel weight (TKW); three-pistil (TP); water absorption (WA); septoria tritici blotch (STB); yellow rust (YR); kernel number of main spike (KNM); days from booting to anthesis (DSB); grain yield (GY); powdery mildew (Pm3).
